# Relationship between Sociodemographics, Dietary Intake, and Physical Activity with Gestational Weight Gain among Pregnant Women in Rafsanjan City, Iran

**Published:** 2015-03

**Authors:** Fatemeh Ebrahimi, Zalilah Mohd Shariff, Seyed Zia Tabatabaei, Mahmood Sheikh Fathollahi, Chan Yoke Mun, Mozhgan Nazari

**Affiliations:** ^1^Department of Nutrition and Dietetics, Faculty of Medicine and Health Sciences, Universiti Putra Malaysia, Serdang, Malaysia; ^2^Department of Social Medicine, School of Medicine, Occupational Environmental Research Center, Rafsanjan University of Medical Sciences, Rafsanjan, Iran; ^3^Department of Public Health, Faculty of Medicine and Health Sciences, Hamadan University of Medical Sciences, Hamadan, Iran

**Keywords:** Dietary intake, Gestational weight gain, Physical activity, Pregnancy, Iran

## Abstract

Gestational weight gain (GWG) is a determinant of health and nutrition of mothers and offspring. However, many factors associated with GWG are not completely understood. The present study assessed the relationship between sociodemographics, dietary intake, and physical activity with GWG in 308 Iranian pregnant women attending government healthcare centres in Rafsanjan city, Iran. Women gained an average of 12.87±3.57 kg during pregnancy while 54% did not gain weight within the Institute of Medicine (IOM)-recommended range. Univariate logistic models showed that gestaional weight gain was related to age, pre-pregnancy body mass index (BMI), energy intake, and sitting time. Cumulative logit model showed positive relationship between age (p=0.0137) and pre-pregnancy BMI (p<0.0001) with GWG. All pregnant women should be counselled on achieving the reccomended GWG to prevent adverse maternal and prenatal outcomes. Pre-pregnancy and gestational nutritional status and physical activity should be emphasized in antenatal care.

## INTRODUCTION

Gestational weight gain (GWG) within the Institute of Medicine (IOM)-recommended range is associated with improved maternal and neonatal outcomes compared to weight gain lower or higher than these recommended levels. Low weight gain during pregnancy is associated with small-for-gestational age, low birthweight, and preterm birth of the baby (1). In contrast, high GWG is related to several complications in mothers, such as hypertension, diabetes, pre-eclampsia, macrosomia, and maternal weight retention postpartum (2). In additon, GWG may also influence body composition in childhood and later life (3,4). Studies have also suggested that excessive GWG is associated with higher fat mass in childhood and greater body mass index (BMI) and fat mass in later adulthood (5,6).

In the developing countries, women generally have a lower GWG than women in the developed countries. Several studies in the United States (1,7) and Europe (8) reported that the mean GWG was in the range of 13-14.2 kg. Ota *et al*. (9) reported that GWG among women (mean age 27.9±5.3 years) in Viet Nam was 12.2 kg. In Iran, Yekta *et al*. (10) and Maddah *et al*. (11) showed that the mean GWG was 8.8 kg and 11.3 kg respectively. The researchers also reported that about 40% of these pregnant women gained within the IOM-recommended level while about 60% had lower or excessive weight gain.

Previous studies have shown that factors, such as pre-pregnancy BMI, height, age, number of prenatal visits, income, smoking, and infant's sex are associated with GWG (12–14). Despite the important contribution of maternal nutritional status and dietary factors to weight gain during pregnancy, only a few studies (15,16) have determined the influences of these factors on maternal weight gain while most studies have focused on the relationship between intake of nutrients and birth outcomes (3,17). At present, data on sociodeomographics, nutrient intakes, and physical activity associated with GWG are insufficient, particularly in many developing countries. Recognizing that information on these modifiable risk factors will be useful in developing appropriate strategies to encouraging adequate GWG, the present study aimed to determine factors asssociated with GWG among Iranian pregnant women.

## MATERIALS AND METHODS

### Subjects

This cross-sectional study was conducted in seven healthcare centres in Rafsanjan city located in the southeast of Iran. The lists of pregnant women were obtained from all health centres. Pregnant women were screened based on the inclusion criteria of Iranian citizen, aged between 18 and 35 years, in the third trimester of pregnancy, who had singleton pregnancy. Out of 860 pregnant women in these healthcare centres, 520 met the selection criteria but only 308 volunteered to participate in this study.

### Measurements

A set of pretested questionnaire was used in assessing sociodemographic background (women's age, education level, and household income), dietary intake, and physical activity of pregnant women.

#### Gestational weight gain

Pre-pregnancy weight was obtained from the prenatal record. The pre-pregnancy weights were measured and recorded by midwives based on the women's visits to the healthcare centres for family planning and medical follow-ups. The weight measured within 1-2 month(s) prior to pregnancy-booking was used as pre-pregnancy weight. To cross-check the recorded weight, the women were also asked to report pre-pregnancy weight. There was a high correlation between these two measures (r=0.93). Height for each woman was measured twice to the nearest 0.1 cm, using Seca bodymeter, and the average of two measurements was used in analysis. Maternal weight was measured in each healthcare centre in the last prenatal visit (38-40 weeks). Weight was measured twice and to the nearest 0.1 kg, using a Seca digital scale. All pregnant women were weighed in light clothes and without shoes. Total pregnancy weight gain was then estimated by subtracting the pre-pregnancy weight from the last-measured weight before delivery. The total weight gain of women during pregnancy were then categorized according to the recommended level of gestational weight gain as per the Institute of Medicine (2009) based on pre-pregnany BMI classifications (18) (Table 1).

#### Physical activity

The present study used the short version of the International Physical Activity Questionnaire (IPAQ) that has been used in both non-pregnant and pregnant populations to estimate physical activity level, namely mild, moderate and vigorous physical activity (19–21). Each pregnant woman was assisted to recall her physical activities in the last seven days. For each type of activity, the frequency and duration were assessed in days per week and minutes or hours per day respectively. Physical activity level, sitting time, and metabolic equivalent (MET) of physical activity were then determined using the guidelines for data processing and analysis of the IPAQ (22).

#### Dietary intake

Two days’ 24-hour recalls were obtained from the respondents, and the reported estimates were based on the average of two recalls. Women were individually interviewed on their intakes of food and beverages. All portion-sizes were described in household measures (cup, spoon, plate, glass), and food album containing coloured pictures (in different portion-sizes) was used for improving the accuracy of the recalls. Portion-sizes of consumed foods were then converted to grammes (23). Dietary data were analyzed for energy and nutrients, using modified Nutritionist IV software [Nutritional Database Manager 4.0.1, Nutritionist IV (First Data Bank, USA), version 3.5.2]. Intake adequacy was determined according to Dietary Recommended Intakes of United States.

**Table 1.** Recommended weight gain during pregnancy

Pre-pregnancy body mass index (BMI)	Recommended weight gain
Underweight (BMI less than 18.5)	28 to 40 lb (12.4 to 18 kg)
Normal weight (BMI 18.5 to 24.9)	25 to 35 lb (11.5 to 16 kg)
Overweight (BMI 25 to 29.9)	15 to 25 lb (7 to 11.5 kg)
Obese (BMI 30 or greater)	11 to 20 lb (5 to 9 kg)

Source: Institute of Medicine (2009)

### Ethical approval

This study was approved by the Medical Research and Ethics Committee, Faculty of Medicine and Health Sciences, Universiti Putra Malaysia and Rafsanjan University of Medical Sciences, Iran. Permission was also obtained from principals in each healthcare centre prior to the study. Written informed consent was obtained from all pregnant women prior to data collection.

### Data analysis

Data were analyzed using Statistical Package for Social Sciences (version 16.0); (SPSS Inc., Chicago, IL, USA) and SAS (version 9.1) for Windows (SAS Institute Inc., Cary, NC, USA) software. Descriptive statistics were computed for all variables. Three separate binary logistic regression models were developed to examine the effect of independent factors associated with having inadequate vs adequate, inadequate vs excessive, and adequate vs excessive weight gain. The relationship between maternal characteristics with GWG (inadequate, adequate, and excessive weight gain) was also examined using cumulative logit model for ordinal responses (24) using the ‘Proc Logistic’ with ‘Link=Clogit’ in SAS (version 9.1) (SAS Institute Inc., Cary, NC, USA). The associations of independent predictors with GWG model (binary logistic and cumulative logit models) were expressed as odds ratios (ORs) with 95% confidence interval (CI). A statistical probability level of p<0.05 was considered significant.

## RESULTS

Maternal characteristics are shown in Table 2. The mean age of women was 26.61±4.71 years, and nearly half (49%) of the women had high school education. The mean number of pregnancy was 1.70±0.94 while more than half (54.5%) of the women had their first pregnancy. About 73% of the women were from low-income households as defined by households with monthly incomes of US$ 500 or less.

Based on pre-pregnancy BMI, 4.2% of the women were underweight, 54.2% had normal weight, 33.1% were overweight, and 9.4% were obese. The women gained an average of 12.87±3.57 kg during pregnancy. The mean total weight gain for underweight and obese women was 14.74±3.77 kg and 11.76±3.28 kg respectively. About 46% of the women gained weight within the recommended range. More than half of the women (54%) had either inadequate or excessive weight gain. For underweight and normal-weight pregnant women, 23.1% and 18.3% had excessive weight gain. However, for overweight and obese women, a higher proportion (52% and 75.9%) gained more than the recommended range.

Pregnant women had either low (66.6%) or moderate (33.4%) level of physical activity. The mean time spent in sitting was the highest (323.21±122.02 minutes/day). Majority (71.4%) of the women did not meet the DRI (Dietary Reference Intake) for energy intake in the third trimester of pregnancy. In addition, a high proportion of these women did not meet the DRI for vitamin A (65.3%), vitamin D (99.1%), magnesium (82.8%), calcium (53.6%), iron (87.2%), zinc (78.9%), and folate (98.1%).

Table 3 indicates the logistic regression models for factors relating to inadequate, adequate, and excessive GWG. The first model shows that women with inadequate weight gain, compared to the adequate group, had lower age (OR=0.892, 95% CI 0.806-0.987), pre-pregnancy BMI (OR=0.812, 95% CI 0.723-0.912), and sitting time (OR=0.997, 95% CI 0.994-0.999). The second model indicates that women with inadequate weight gain, compared to excessive group, had lower pre-pregnancy BMI (OR=0.629, 95% CI 0.532-0.745) and energy intake (OR=0.998, 95% CI 0.997–0.999) than excessive group. In the third model, pregnant women in the group of adequate weight gain had lower pre-pregnancy BMI (OR=0.840, 95% CI 0.777-0.909) compared to the group of excessive weight gain. The cumulative logit model (Table 4) shows the following factors to be significantly associated with increasing GWG: age (OR=1.083, 95% CI 1.016–1.153) and pre-pregnancy BMI (OR=1.507, 95% CI 1.182-1.349; p<0.0001). In other words, pregnant women with higher age and higher pre-pregnancy BMI were more likely to have higher GWG.

## DISCUSSION

Gestational weight gain (GWG) is an important determinant of perinatal outcomes (25,26). The present study found that the mean GWG was higher compared to 8.8 kg and 11.3 kg reported by Yekta *et al*. (10) and Maddah *et al*. (11) for Iranian pregnant women in the north (Rasht) and northwest (Urmia) of Iran. The different findings could be related to the better socioeconomic status (educational level and household income) of pregnant women in the present study compared to the women in the aforementioned studies. The higher socioeconomic status is then translated to better access to food quantity and quality, antenatal care as well as health and nutrition information (27). Consistent with previous studies (10,11), we showed that the mean total GWG was the highest among underweight women but excessive GWG was more common among overweight and obese women. Both inadequate and excessive weight gain among pregnant women should receive special attention as they pose a threat to mothers as well as infants.

**Table 2.** Background characteristics of pregnant women (N=308)

Characteristics	No. (%)	Mean±SD
Age (completed years)		26.61±4.71
18-24	113 (36.7)	
25-29	111 (36.0)	
30-35	84 (27.3)	
Educational level (years of schooling)		10.99±3.15
Low education^a^	93 (30.2)	
High school diploma	151 (49.0)	
University	64 (20.8)	
Household income (US$)		445.29±299.70
≤500	224 (72.7)	
>500	84 (27.3)	
Number of pregnancy		1.70±0.94
1	168 (54.5)	
≥2	140 (45.5)	
Pre-pregnancy weight (kg)		63.54±10.86
Height (m)		1.62±0.05
Pre-pregnancy BMI^b^ (kg/m²)		24.62±4.05
Underweight (BMI <18.5)	13 (4.2)	
Normal weight (BMI 18.50 to 24.99)	164 (53.2)	
Overweight (BMI 25 to 29.99)	102 (33.1)	
Obese (BMI ≥30)	29 (9.4)	
Pregnancy weight gain		
Total weight gain (All) (kg)		12.87±3.57
Inadequate weight gain	59 (19.2)	
Adequate weight gain	141 (45.7)	
Excessive weight gain	108 (35.1)	
Total weight gain (Underweight) (kg)		14.74±3.77
Inadequate weight gain	3 (23.1)	
Adequate weight gain	7 (53.8)	
Excessive weight gain	3 (23.1)	
Total weight gain (Normal) (kg)		13.21±3.54
Inadequate weight gain	55 (33.5)	
Adequate weight gain	79 (48.2)	
Excessive weight gain	30 (18.3)	
Total weight gain (Overweight) (kg)		12.41±3.64
Inadequate weight gain	1 (0.9)	
Adequate weight gain	48 (47.1)	
Excessive weight gain	53 (52.0)	
Total weight gain (Obese) (kg)		11.76±3.28
Inadequate weight gain	0	
Adequate weight gain	7 (24.1)	
Excessive weight gain	22 (75.9)	
Physical activity level		
Low	205 (66.6)	
Moderate	103 (33.4)	
High	0	
Total MET^c^		554.06±375.97
Sitting (minutes/day)		323.21±122.02
Dietary intake		
Calorie (kcal)		2271±526
Carbohydrate (g)		310.55±101.96
Protein (g)		78.23±23.73
Fat (g)		70.27±25.84
Vitamin A (RE)		1072.48±1810.74
Vitamin C (mg)		120.22±96.01
Vitamin D (μg)		1.10±1.51
Magnesium (mg)		242.31±170.92
Calcium (mg)		968.51±363.05
Sodium (mg)		1735.83±663.69
Iron (mg)		17.63±9.90
Zinc (mg)		9.62±13.42
Folate (μg)		217.57±141.50

^a^Low education included ‘No formal schooling’ and ‘Primary schooling’

^b^BMI=Body mass index

^c^MET=Metabolic equivalent of physical activity (minutes/week)

Previous studies have reported conflicting findings on the relationship between age and GWG. In a comparative study of older (≥35 years old) and younger (25-29 years old) pregnant women, Prysak *et al*. (28) found that older women had significantly lower mean GWG than younger women. Similarly, Olafsdottir *et al*. (3) reported that women gaining excessive gestational weight were significantly younger than women gaining less weight. Other studies have shown that older women tend to gain more weight during pregnancy (1,13,29). A study by Rodrigues *et al*. (14) showed that, for each increase of one year in the woman's age, there was an increase of 0.631 kg in gestational weight. The present study also found that women's age was positively associated with GWG but this relationship was confined to older women (30-35 years) who were more likely to have adequate than inadequate weight gain. Perhaps, older women in our study were more aware of adequate weight gain during pregnancy through improved dietary intake compared to younger women.

Consistent with previous studies (29–31), we showed that pre-pregnancy BMI is an important factor in determining GWG among pregnant women. Wiesman *et al*. (29), in a study among 103 pregnant women, reported that being overweight substantially increases the odds of exceeding the IOM guidelines compared to normal preconception weight category [adjusted odds ratio (AOR) 2.84]. Being overweight or obese before pregnancy or having low pre-pregnancy BMI was a risk factor for respectively excessive or insufficient weight gain during pregnancy. This emphasizes the importance of specific counselling on weight gain recommendations for underweight and overweight women. Insufficient weight gain is a reason for concern due to the possibility of increased low birthweight, prematurity, longer hospital stay and, consequently, higher health-related costs (4). Excessive gestational weight gain should also be considered a public-health problem as it has been shown to contribute to postpartum weight retention and, consequently, increased rates of obesity in women, which is a risk factor of cardiovascular disease, diabetes, and several types of cancer (3,32). Therefore, women with higher pre-pregnancy BMI should be more concerned about gaining weight during pegnancy due to risk of postpartum weight retention and obesity. It is essential for healthcare providers to advise mothers during antenatal care visits on appropriate gestational weight gain through healthy dietary and lifestyle habits to prevent further increase in weight postpartum.

**Table 3.** Adjusted odds ratios (OR) for factors associated with inadequate, adequate, and excessive gestational weight gain

Variable	Gestational weight gain (GWG) OR (95% CI)		
	Inadequate vs Adequate	Inadequate vs Excessive	Adequate vs Excessive
Age (years)	0.892 (0.806-0.987)*	0.892 (0.779-1.000)	0.964 (0.893-1.040)
Pre-pregnancy BMI (kg/m^2^)	0.812 (0.723-0.912)**	0.629 (0.532-0.745)***	0.840 (0.777-0.909)***
Education (years)	0.997 (0.876-1.134)	0.987 (0.849-1.147)	0.948 (0.861-1.043)
Number of pregnancy	1.573 (0.859-2.882)	1.358 (0.628-2.938)	1.061 (0.666-1.691)
Household income (US$)	1.001 (1.000-1.002)	1.002 (1.000-1.003)	1.000 (0.999-1.001)
Dietary intake			
Energy (kcal)	0.999 (0.998-1.000)	0.998 (0.997-0.999)*	1.000 (0.999-1.001)
Fat (g)	1.002 (0.987-1.017)	1.002 (0.983-1.021)	0.996 (0.983-1.009)
Protein (g)	0.989 (0.974-1.004)	0.989 (0.971-1.007)	1.003 (0.991-1.014)
Carbohydrate (g)	1.001 (0.996-1.006)	1.001 (0.996-1.007)	0.997 (0.993-1.001)
Physical activity			
Total MET	1.001 (1.000-1.002)	1.001 (1.000-1.002)	1.000 (0.992-1.001)
Sitting time (minutes/day)	0.997 (0.994-0.999)*	0.999 (0.995-1.002)	1.002 (0.999-1.004)

*p<0.05

**p<0.001

***p<0.0001

Findings on the relationship between physical activty and gestational weight gain have been somewhat inconsistent. A review by Siega-Riz *et al*. (33) found that women who continued endurance exercise at or near pre-pregnancy stages gained less weight than those who stopped exercise. In a study on effects of running on weight gain during pregnancy, maternal weight gain was similar among women who continued to run compared to the control group who did not continue to run during pregnancy (34). Streuling *et al*. (35) suggested that physical activity during pregnancy might be successful in restricting excessive weight gain. We showed that, although sitting time was positively associated with gestational weight gain in the binary logistic model for inadequate vs adequate weight gain, the association did not persist in the cumulative logit analysis. However, several studies have reported positive relationship between sitting time with overweight, obesity, and weight gain in non-pregnant population (36,37).

A few studies have examined the relationship between nutrient intake and weight gain, and the majority have focused primarily on other determinants (age, education, income, working status) of inadequate weight gain (32,38–40). Although we expected some nutrient intakes to predict pregnancy weight gain in the cumulative logit model, only energy intake was significant in binary logistic regression (between inadequate vs excessive group). There was no significant difference in macronutrients intake between the weight gain groups; however, percentage of energy intake from protein was higher among excessive group compared to inadequate group in post-hoc test (ANOVA) (data not shown). Olafsdottir *et al*. (3) reported that total energy intake was the highest among the group of excessive weight gain. They showed that absolute energy intake also correlated with maternal weight gain (*r*=0.112, p=0.024), and the total amounts of protein, fat, and carbohydrate contributed to this weight gain. Earlier studies (41,42) had indicated that maternal protein intake is positively associated with pregnancy weight gain. As excess calorie intake is a potential risk of excessive weight gain during pregnancy, pregnant women who are at risk of excessive weight gain may require assistance in adopting healthy dietary and lifestyle behaviours.

**Table 4.** Factors associated with gestational weight gain (Cumulative Logit Model for Ordinal Responses)

Variable	OR	95% CI	p value
Age (years)	1.083	(1.016-1.153)	0.0137
Pre-pregnancy BMI (kg/m^2^)	1.507	(1.182-1.349)	<0.0001
Education (years)	1.042	(0.963-1.128)	0.3019
Number of pregnancy	0.762	(0.519-1.117)	0.1637
Household income (US$)	1.000	(0.999-1.000)	0.2213
Dietary intake			
Energy (kcal)	1.000	(1.000-1.001)	0.2543
Fat (g)	1.002	(0.991-1.012)	0.7449
Protein (g)	1.002	(0.993-1.012)	0.6366
Carbohydrate (g)	1.002	(0.998-1.005)	0.3161
Physical activity			
Total MET	1.000	(0.999-1.000)	0.1909
Sitting time (minutes/day)	1.001	(0.999-1.003)	0.4825

### Strengths and limitations

This study had several limitations. First, pre-pregnancy weight was based on weights in the prenatal records. It is likely to be underestimated or overestimated and could result in an inaccurate estimation of GWG. Nevertheless, there was a high correlation between the recorded weights and pre-pregnancy weights as reported by mothers. Second, the sample-size of underweight and obese women in the study was small, and purposive sampling technique was used. In addition, women were from a limited geographic region in the southeast of Iran, and only urban pregnant women were included in the study. These factors could limit the study's generalizability to pregnant women in Iran. Third, the IPAQ and 24-hour dietary recall relied on individuals’ understanding of the instruments and their ability to recall food intakes and physical activity accurately. For example, the women might experience difficulty to distinguish between moderate and vigorous activities. Despite these limitations, this study was the first to be conducted among pregnant women in Rafsanjan city in Kerman province in the southeast of Iran and provide evidence of weight gain and related factors among pregnant women

### Conclusions

This study found that, although approximately half of the pregnant women had adequate GWG, there was also a high proportion of pregnant women gaining excessive weight, particularly those who were overweight and obese. In addition, there was positive relationship between maternal characteristics, such as age, pre-pregnancy BMI, energy intake, and sitting time with gestational weight gain. As inadequate or excessive prenatal weight gain could have adverse effects on both mother and child, there is a need to inform pregnant women to gain appropriate amount of weight during pregnancy. Identifying women at risk of inadequate or excessive weight gain earlier in pregnancy could be a challenge but efforts to address modifiable risk factors (e.g. diet and physical activity) could prevent women from having at-risk GWG.

## ACKNOWLEDGEMENTS

The authors acknowledge the support of all mothers, midwives, and staff of the healthcare centres during data retrieval for the study.

**Conflict of interest:** Authors declare no conflicts of interest
